# Effect of Annealing Temperature on the Microstructure and Mechanical Properties of High-Pressure Torsion-Produced 316LN Stainless Steel

**DOI:** 10.3390/ma15010181

**Published:** 2021-12-27

**Authors:** Yuanyuan Dong, Zhe Zhang, Zhihai Yang, Ruixiao Zheng, Xu Chen

**Affiliations:** 1 Science and Technology on Reactor System Design Technology Laboratory, Nuclear Power Institute of China, Chengdu 610213, China; sshuimus@163.com (Y.D.); yangzhisea@163.com (Z.Y.); 2 School of Chemical Engineering and Technology, Tianjin University, Tianjin 300350, China; xchen@tju.edu.cn; 3 Zhejiang Institute of Tianjin University, Ningbo 315201, China; 4 Key Laboratory of Aerospace Advanced Materials and Performance of Ministry of Education, School of Material Science and Engineering, Beihang University, Beijing 100191, China; zhengruixiao@buaa.edu.cn

**Keywords:** 316LN stainless steel, ultrafine grains, high-pressure torsion, mechanical properties, deformation mechanism

## Abstract

316LN stainless steel is a prospective structural material for the nuclear and medical instruments industries. Severe plastic deformation (SPD) combined with annealing possesses have been used to create materials with excellent mechanical properties. In the present work, a series of ultrafine-grained (UFG) 316LN steels were produced by high-pressure torsion (HPT) and a subsequent annealing process. The effects of annealing temperature on grain recrystallization and precipitation were investigated. Recrystallized UFG 316LN steels can be achieved after annealing at high temperature. The σ phase generates, at grain boundaries, at an annealing temperature range of 750–850 °C. The dislocations induced by recrystallized grain boundaries and strain-induced nanotwins are beneficial for enhancing ductility. Moreover, microcracks are easy to nucleate at the σ phase and the γ-austenite interface, causing unexpected rapid fractures.

## 1. Introduction

316LN austenitic stainless steel has excellent mechanical properties and good resistance to pitting corrosion at high temperatures, so it is a prospective structural material for nuclear equipment, surgical instruments and orthopedic implants [1,2]. Compared with conventional 316L stainless steel, the addition of nitrogen could stabilize austenite and introduce solid solution hardening. Furthermore, 316LN steels have much higher yield strength and tensile strength than 316L steels [3]. However, 316LN stainless steel has a relatively low yield strength (250–400 MPa); therefore, it is imperative to enhance the strength for improving equipment safety.

Although nanocrystalline (NC) or ultrafine-grained (UFG) materials produced by thermo-mechanical processes or severe plastic deformation (SPD) processes demonstrated a relatively high strength, their lack of ductility restricts their real applications in the industry [4,5,6,7,8]. Therefore, there is a strong interest in the applications of SPD combined with annealing processes to produce stainless steels, having a good balance of high strength and high ductility [9,10,11,12,13,14,15,16]. An enhanced uniform elongation in a UFG 316LN stainless steel produced by thermo-mechanical process was attributed to strain-induced nanoscale twins (NTs) [9]. Li et al. [10] produced heterogeneous lamella-structured (HLS) 316L steels by cold rolling and annealing. It was found that high back stress was induced in the heterostructure interfaces, resulting in superior high strength and high ductility. Zheng et al. [11] also produced hierarchical structured 316L steels by powder metallurgy and subsequent thermo-mechanical processes. The improved yield strength was related to the refined grains and hetero-deformation-induced strengthening. Simultaneously, uniform elongation can be enhanced by accumulated dislocations, defects and NTs. Zhang et al. [12,13,14] developed stainless steels with harmonic structures by mechanical milling and powder metallurgy processes. The continuous network UFG structure is beneficial for realizing the steels, having a good balance of high strength and large ductility [12,13,14]. In addition, high-entropy alloys, having a hierarchical nanolaminate structure produced by thermo-mechanical processes, demonstrated superior strength–ductility synergy. The unique mechanical properties were related to the bidirectional transformation induced plasticity (B-TRIP) and the twinning-induced plasticity (TWIP) effect [15,16].

It is well known that phase transformation and precipitation is often generated during annealing; this is due to the complicated alloy element compositions in stainless steels, which play an important influence on recrystallization and mechanical properties. Therefore, the phase transformation and precipitation of stainless steels during thermo-mechanical processes and SPD possess were investigated extensively [17,18,19,20,21,22,23,24]. It was reported that strain-induced martensite transformation and martensite to austenite reversion by annealing promoted the generation of ultrafine grains [17]. Martensite thermo-mechanical treatment was used to produce a bimodal NC/UFG structured stainless steels having good strength and ductility. It was found that ferrite–austenite phase transformation, which occurred during annealing, could produce austenite/ferrite lamellar structures in a hot-rolled 304H stainless steel [18]. Moreover, accompanied with recrystallization, many types of precipitates also tend to form during annealing [19]. Carbide precipitation was identified during recrystallization of cold-rolled 201 steels [20]. CrN nitrides were also formed in UFG 301LN steels to increase the yield strength [21]. Moreover, it is difficult to prevent the precipitation of the sigma phase in stainless steels, as they have high Cr content [23,24]. Although precipitation strengthening contributes to strength, the shape and size of precipitates affects ductility due to their inconsistent deformation. Therefore, it is necessary to reveal microstructure evolution, i.e., grain recrystallization, phase and precipitation, in the annealed SPD-produced stainless steels, as well as its influence on mechanical properties.

Compared with other SPD or thermo-mechanical processes, high-pressure torsion (HPT) process could impact extremely high strain (~300) on bulk materials, which is an important method for producing NC bulk materials [25,26,27,28,29]. Microstructure evolution, phase transformation and mechanical properties of HPT-produced stainless steels were investigated at room temperature [2,30]. It was reported that γ-austenite in 316L steels converted to the ε-martensite initially, and ε-martensite continuously converted to α′-martensite as the applied HPT strain increased. By contrast, nitrogen could stabilize martensite transformation in 316L steels [31]. Namely, γ-austenite 316LN steels converted to both ε-martensite and α′-martensite with increasing HPT strain [2]. Moreover, the effects of temperature on the microstructure and the mechanical properties of HPT-produced materials were also investigated in [32,33]. Usually, HPT-produced materials have inhomogeneous microstructure at room temperature, which contains nanograins, distorted boundaries and intragranular defects, etc. When the HPT temperature elevated, deformation twinning and dislocation glide were dominant instead of phase transformation. The higher temperature promoted high angle boundary formation and grain recrystallization. 

As mentioned, the annealing temperature has a great influence on microstructure evolution and mechanical properties of SPD-produced materials. In general, the annealing process can promote grain recrystallization, resulting in the reduction in strength [34,35,36,37]. However, unexpected strengthening was observed in the annealed HPT-produced 316L steels [31]. The HPT-produced 316L steels were annealed at temperature ranging from 300–650 °C. It is interesting to note that the annealed HPT 316L steels showed the higher strength and hardness than HPT 316L steels. Moreover, the *G* phase was generated at the NC grain boundaries. Therefore, it is of interest to investigate the effect of annealing temperature on the microstructure and the mechanical properties of HPT-produced materials. Until now, the understanding of annealing temperature’s effect on grain recrystallization, precipitation and mechanical properties of HPT-produced 316LN steels are still inadequate. Therefore, a series of UFG 316LN stainless steels were fabricated using HPT combined with an annealing process in the present work. The evolution of grain structure and precipitates at different annealing temperatures were investigated. The relationship between microstructure and mechanical properties were clarified. Finally, deformation mechanism and failure mechanism of the annealed UFG 316LN steels were analyzed.

## 2. Materials and Methods

The chemical compositions of the applied 316LN austenitic stainless steel are listed in Table 1. The effects of HPT revolution on the grain structure, phase evolution and the mechanical properties at room temperature were reported in the previous work [2]. The HPT process was performed at pressure of 5 GPa with a constant rotation speed of 0.5 rpm at room temperature. NC 316LN steel was achieved after HPT for 5 revolutions (HPT-5N). Compared with 316L steel, 316LN steel has lower stacking fault energy and nitrogen atoms can promote dislocation accumulation. Therefore, the nanostructure and martensite transformation tend to become saturated after 5 revolutions. Subsequently, the HPT-produced steels were annealed at the temperatures ranging from 700 to 900 °C, for 30 min in a salt bath. Finally, the annealed steels were quenched in the cold water.

The phase transformation of the produced samples was examined by X-ray diffraction with CuKα radiation (XRD, Bruker, Billerica, MA, USA). Precipitates and grain structure were observed by a field emission scanning electron microscope (FE-SEM, FEI Apreo S, Brno, Czech Republic) operating at 20 kV. Grain size and volume fraction of precipitates were calculated from SEM pictures by image analysis. The samples were mechanical polished by SiC paper and colloidal silica suspension (OP-S, Struers, Rodovre, Denmark). A solution of 45% HCl, 15% HNO_3_ and 40% CH_3_OH was used to etch the samples. Moreover, transmission electron microscopy (TEM, FEI Tecnai G2 F20 S-TWIN, Brno, Czech Republic) with an energy dispersive spectrometer (EDS, Edax, Pleasanton, CA, USA) analysis were also used to analyze precipitates under 200 kV. 

The HPT-produced samples had a diameter and a thickness of 10 mm and ~0.8 mm, respectively. According to Reference [2], the tensile specimens were cut by wire cutting. The gauge length of the specimens was 2 mm, and the cross-section area was 1 mm × 0.8 mm. The tensile properties were measured by a uniaxial fatigue testing machine (CARE, IBTC-5000) under displacement control. The nominal strain rate was approximately 8.33 × 10^−4^/s. A non-contact CCD extensometer system was used to measure the displacement of the gauge area. At least three samples were measured for reproducibility. The strain hardening rate (*dσ⁄dε*) was determined from true stress–strain curves.

## 3. Results and Discussion

### 3.1. Effect of Annealing Temperature on the Microstructure of HPT-Produced 316LN Steels

**Figure 1:**
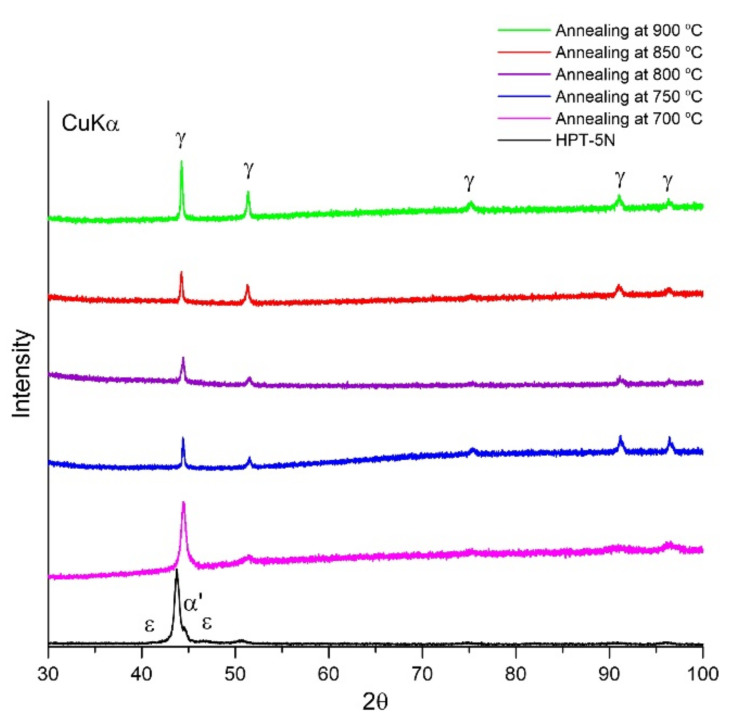
XRD results of HPT-produced 316LN steels annealed at different temperatures. HPT-5N: HPT for 5 revolutions.

**Figure 2:**
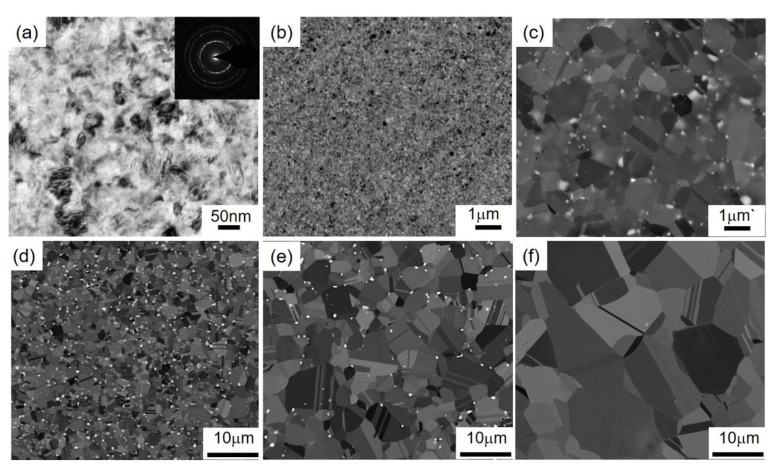
Microstructure of HPT-produced 316LN steels annealed at different temperatures: (**a**) HPT-5N; (**b**) 700 °C annealing; (**c**) 750 °C annealing; (**d**) 800 °C annealing; (**e**) 850 °C annealing; (**f**) 900 °C annealing.

Figure 1 shows XRD results of the HPT-5N 316LN steels annealed at different temperatures. As reported, HPT-5N 316LN steels contain γ-austenite, ε-martensite and α′-martensite. Unlike 316L stainless steels, ε-martensite cannot transform completely to α′-martensite in 316LN steels by the HPT process [2]. As indicated in Figure 1, all annealed steels have a γ-austenite structure, indicating that both ε-martensite and α′-martensite reverse to γ-austenite during annealing process. The microstructure evolution of HPT-5N 316LN steels at different annealing temperatures are shown in Figure 2. The diffraction ring of HPT-5N 316LN stainless steel is continuous, which indicates that the structure is not homogenous. Nanograins, distorted boundaries and intragranular defects exist in the HPT-5N 316LN stainless steel (see Figure 2a). Followed by annealing, it can be observed from Figure 2b–f that the recrystallized equiaxed grains generate, and that grain size increases gradually with increasing annealing temperature. Moreover, some particles generate at grain boundaries in the steels annealed at a temperature range of 750–850 °C.

**Figure 3:**
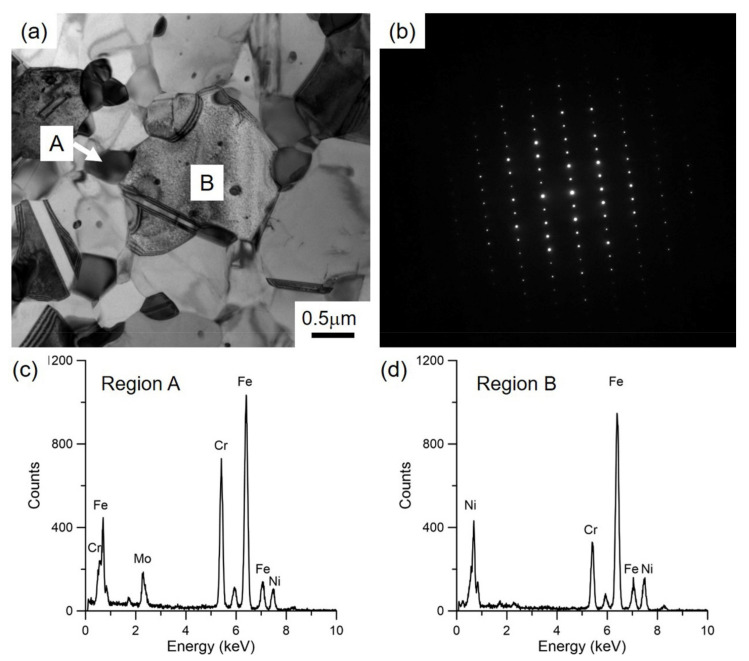
TEM observations of UFG 316LN stainless steel annealed at 800 °C: (**a**) morphology; (**b**) diffraction pattern; (**c**) chemical compositions of precipitations; (**d**) chemical compositions of base materials.

Figure 3 shows the difference of morphology and chemical compositions between precipitates and 316LN steel matrix annealed at 800 °C. As shown in Figure 3a, the precipitates have globular structure, and particle size ranges from 20 nm to 500 nm. As indicated in Figure 3a,b, the annealed 316LN steel matrix has an equiaxed UFG structure with γ-austenite. Figure 3c shows the chemical compositions of the precipitates, which mainly contain Fe, Cr and Mo. By contrast, the 316LN steel matrix is mainly composed of Fe, Cr and Ni in Figure 3d. According to the chemical compositions of typical precipitations in austenitic stainless steels, these Fe–Cr–Mo precipitations are termed as the σ phase in the annealed 316LN steels [23,24].

**Figure 4:**
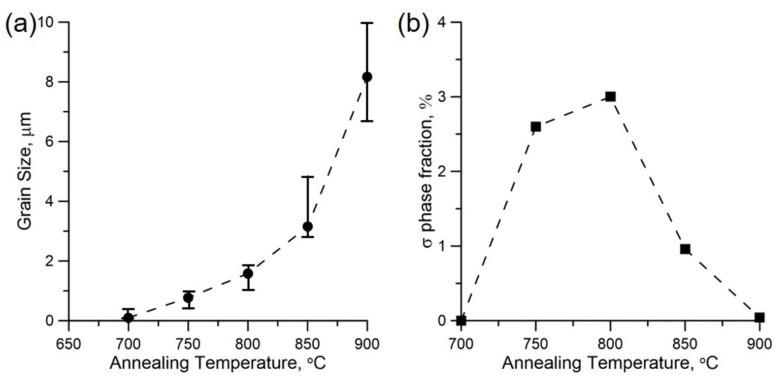
(**a**) Grain size; (**b**) σ phase volume fraction of HPT-produced 316LN steels under different annealing temperatures.

Figure 4a,b quantitatively summarize the effect of annealing temperature on the evolution of grain size and the volume fraction of the σ phase, respectively. The steel annealed at 700 °C has an average grain size of approximately 0.1 μm. As expected, the grain size increases with increasing annealing temperature. The recrystallized grain size increases from 0.8 to 8.2 μm as the temperature increases from 750 to 900 °C. Simultaneously, the effect of annealing temperature on σ phase formation is also significant. The σ phase initially occurs at 750 °C and its volume fraction is approximately 2.6%. The maximum volume fraction of the σ phase (approximately 3%) appears in the steel annealed at 800 °C. As the temperature continuously increases to 900 °C, the σ phase disappears in the annealed steel, providing a fully γ-austenite structure. 

It was reported that many types of precipitates tend to generate in austenitic stainless steels during annealing due to the complicated alloy elements [19]. According to the time–temperature–transformation (TTT) curves of the σ phase in 316 steels, the σ phase initially appears at a temperature range of 900–950 °C, as well as at annealing times over 8 h [23]. Moreover, carbide and nitride often generate earlier than the σ phase, so the σ phase initial precipitation time can be delayed by the increase in the nitrogen content. Thus, it was reported that the cellular precipitation of Cr_2_N appeared, rather than the σ phase, in CG 316LN steels annealed at 900 °C [38]. Moreover, the *G* phase also appeared in the HPT-produced 316LN steels annealed at a temperature range of 300–650 °C [31]. By contrast, as illustrated in Figure 2c–e, it is noteworthy that the σ phase initially appears from 750 °C to 850 °C within 30 min in our investigations. Namely, both annealing temperature and precipitation time for σ phase formation are lower than that of CG bulk 316LN steels. Therefore, it is concluded that the refined grain boundary by HPT process could accelerate σ phase formation.

### 3.2. Mechanical Properties of HPT-Produced 316LN Steels after Annealing

**Figure 5:**
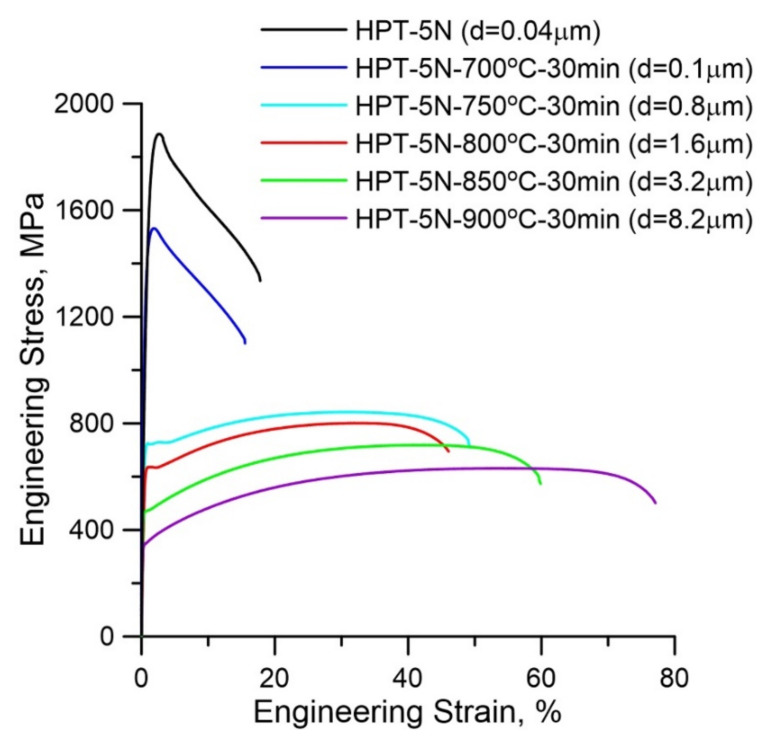
Tensile results of HPT-produced 316LN stainless steels after annealing.

**Figure 6:**
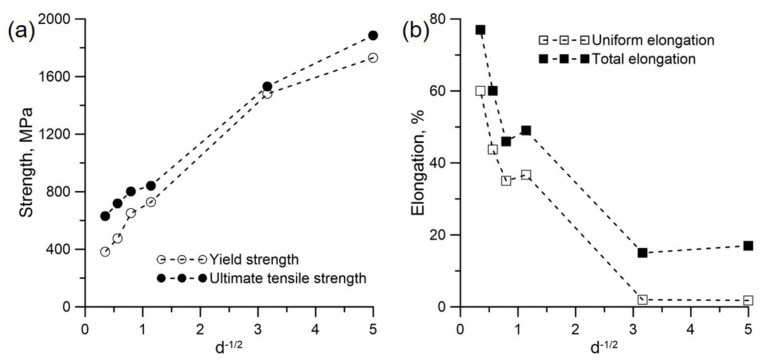
Effect of grain size on (**a**) strength and (**b**) elongation.

Figure 5 shows the tensile results of the HPT-produced 316LN steels subjected to different annealing temperatures. It can be seen from Figure 5 that both the yield strength and the ultimate tensile strength (UTS) of the initial NC 316LN steel were extremely high (>1700 MPa), whereas the uniform elongation was only 1.8%. By contrast, the steel annealed at 700 °C had lower strength but similar elongation. It is noted that elongation was highly improved in the steel annealed at 750 °C. However, it is interesting to note that both strength and elongation decreased in the steel annealed at 800 °C. Subsequently, the strength decreased and the elongation increased with increasing annealing temperature. The effects of grain size on the strength and elongation of the annealed 316LN steels are summarized in Figure 6. Figure 6a,b present the change of strength and ductility with respect to the inverse square root of average grain size (*d*^−1/2^), respectively. As illustrated in Figure 6a, the yield strength increases almost linearly with an increase in *d*^−1/2^, which agrees with Hall–Petch relationship. Therefore, it indicates that the reduction in strength of the annealed 316LN steels is mainly attributed to grain coarsening. Although some σ phase precipitates exist in the annealed steels, they have little influence on strength. As indicated in Figure 5 and Figure 6, the strength decreases obviously as the grain size increases to 0.1 μm, but the elongation is similar. As grains size increases to approximately 0.8 μm, the yield strength of the annealed 316LN stainless steel is 727 MPa, which is nearly twice as much as that of CG bulk 316LN steels. Simultaneously, it also keeps a good elongation (~50%). However, unexpected reduction in both strength and elongation is observed in the steel annealed at 800 °C. Subsequently, the continuously increased grain size causes the reduced strength and enhanced ductility.

**Figure 7:**
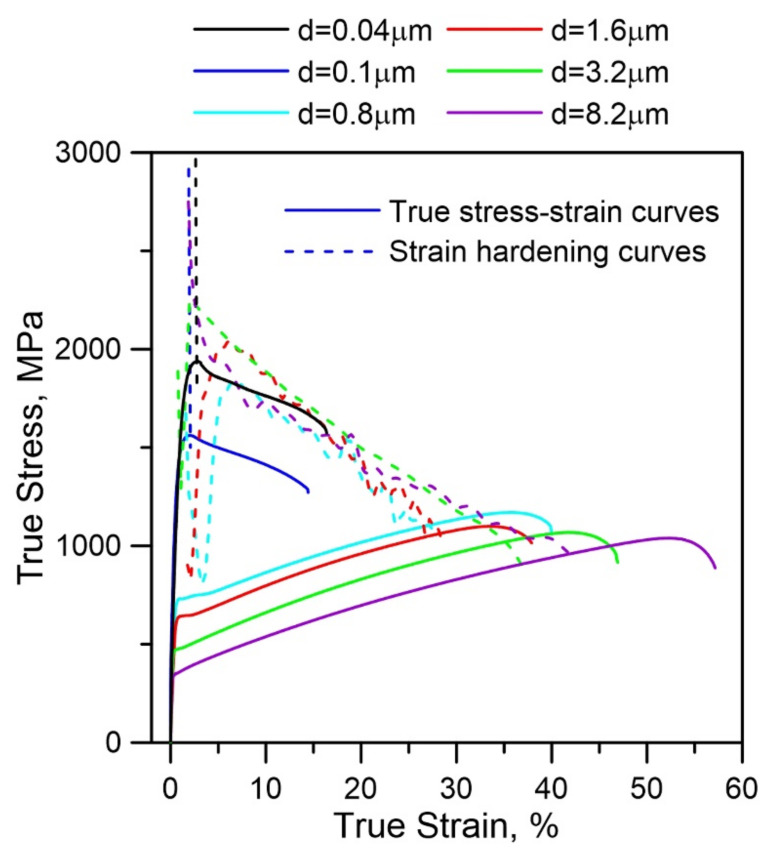
Strain hardening rates of annealed HPT-produced 316LN steels.

As demonstrated in Figure 5, the uniform elongation of the annealed 316LN steel (*d* = 0.8 μm) is evidently improved. It is well known that uniform elongation is always related to strain hardening [11,39]. Therefore, the strain hardening rates of the annealed 316LN steels are shown in Figure 7. The change of strain hardening rates is similar as the grain size is lower than 0.1 μm. Namely, it decreases deeply at the beginning of deformation, which causes the early plastic instability, resulting in the deterioration of uniform elongation. On the contrary, as the grain size reaches 8.2 μm, the strain hardening rate decreases gently, which is identical with conventional CG 316LN steels. However, the tendency of strain hardening rates in the annealed 316LN steels is different as the grain size is over than 0.8 μm. Namely, the rapid strain hardening rate reduction can be seen firstly, but it increases rapidly in the following strain. After that, it decreases gently as similar as conventional CG 316LN steels with continuously increasing elongation. The enhanced additional strain hardening causes the plastic instability delay, resulting in the improved ductility. 

**Figure 8:**
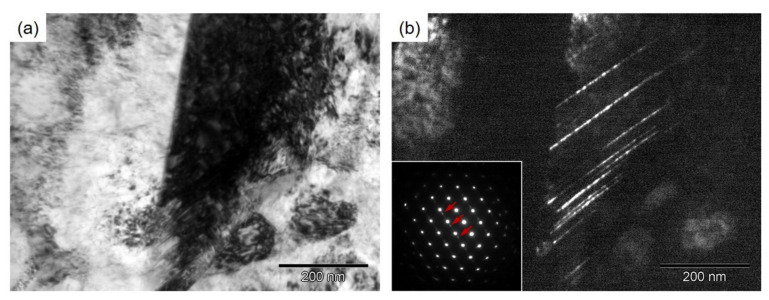
TEM observations of deformed HPT-produced 316LN steel annealed at 750 °C: (**a**) bright field; (**b**) dark field.

The microstructure characteristics of the deformed 316LN steel annealed at 750 °C are presented in Figure 8. Figure 8a,b summarize the representative TEM microstructure of the specimen (*d* = 0.8 μm), stretched to 20%, taken by bright-field and dark-field, respectively. Figure 8a illustrates that the ultrafine grains contain a high density of dislocations. Simultaneously, it is noted that a large number of nanotwins (NTs) with FCC structures are also generated in the ultrafine grains (see Figure 8b). The plastic deformation may change from dislocation slip to deformation twinning in low SFE materials [9]. In addition, many earlier works reported that the refined grain can restrict the strain-induced martensitic transformation, indicating that the grain refinement could enhance the stability of γ-austenite structure [40]. Therefore, it is found that lots of strain-induced NTs are formed in the deformed ultrafine grains of 316LN steels. The competition between the dislocation storage and dynamic recovery plays an important influence on strain hardening rate [41,42]. The recrystallized ultrafine grain boundaries can accumulate much more dislocation compared with nanograin boundaries, which leads to the enhanced strain hardening. Simultaneously, strain-induced NTs within ultrafine grains also hinder dislocation motion effectively, providing an additional strain hardening. Therefore, the enhanced ductility in UFG 316LN steels is attributed to both recrystallized grains and strain-induced NTs.

### 3.3. Fracture Mechanism of Annealed HPT-Produced 316LN Steels

**Figure 9:**
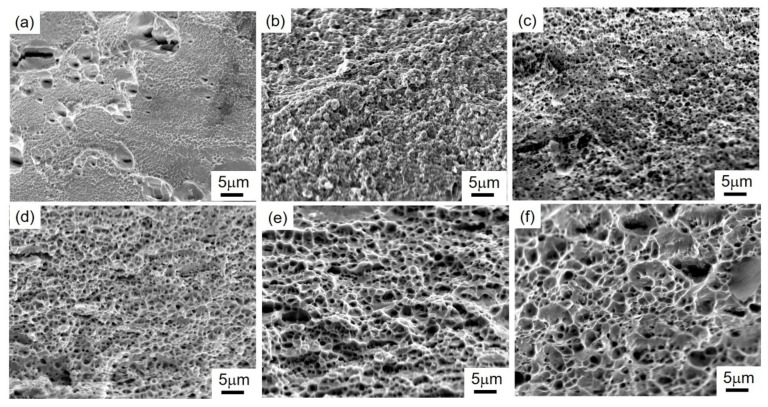
Fracture surface of the annealed UFG 316LN steels: (**a**) HPT-5N; (**b**) 700 °C annealing; (**c**) 750 °C annealing; (**d**) 800 °C annealing; (**e**) 850 °C annealing; (**f**) 900 °C annealing.

The typical fracture morphology of the 316LN steels produced by HPT and annealing process is presented in Figure 9. The NC 316LN steel produced by HPT has a smooth fracture surface with dimples (see Figure 9a). The dimples are shallow, and the size of the dimples is several times larger than the initial grain size. Other NC alloys show a similar phenomenon [43,44]. Moreover, some large cracks form perpendicular to the fracture surface during tensile deformation. The shallow dimples and the large cracks indicate the loss of ductility. Homogeneous dimples are formed by micro void formation and coalescence. By contrast, as shown from Figure 9b–f, relatively rough fracture surface appears in the annealed UFG 316LN steels. Moreover, the dimples size and depth increase with increasing grain size, which indicates the extensive plastic deformation prior to fracture. 

**Figure 10:**
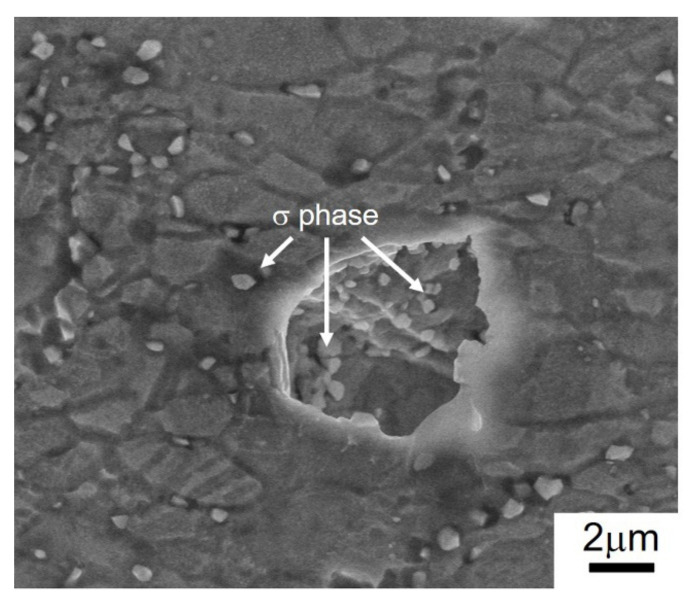
Crack nucleation in UFG 316LN stainless steels annealed at 800 °C.

As shown in Figure 5, the unexpected elongation drop appears in UFG 316LN steel annealed at 800 °C (*d* = 1.6 μm). The profile surface of the deformed steel is analyzed in detail. It can be seen from Figure 10 that microcrack generates near the σ phase particles. As shown in Figure 2d, plenty of σ phase precipitations remain in UFG 316LN steels annealed at 800 °C. It was reported that the σ phase is harder than γ-austenite [24]. Owing to the different ductility between them, microcracks tend to nucleate at the interface of the σ phase and γ-austenite, which is harmful to the elongation. In our present work, it is noted that the σ phase has little influence on the strength but causes losses in ductility. Therefore, the σ phase formation need to be restricted in UFG 316LN steels for improving ductility. As mentioned, σ phase precipitation in steels is related to annealing time and annealing temperature. Thus, it is proposed to restrict σ phase precipitation by reducing annealing time.

## 4. Conclusions

In the present work, HPT-produced 316LN stainless steels were annealed at different temperatures. The microstructure evolution, mechanical properties, as well as their deformation and failure mechanisms, were discussed. Based on this analysis, conclusions are summarized as follows:

(1) Recrystallized UFG structure can be achieved from HPT-produced NC 316LN steels by annealing. The grain coarsening occurs as the annealing temperature elevates from 700 to 900 °C. The σ phase appears at the grain boundary when the annealing is performed between 750 and 850 °C. The refined grain boundary could accelerate σ phase formation.
(2) NC 316LN stainless steels (*d* < 0.1 μm) demonstrate extremely high strength, but the ductility is insufficient. The increased grain size causes the decline of strength and the improvement of elongation.
(3) Strain-induced NTs generate in the ultrafine grains during tensile deformation. The enhanced ductility in UFG 316LN steels is related to the accumulated dislocations by recrystallized grain boundaries and strain-induced NTs.
(4) The annealed UFG 316LN stainless steels demonstrate ductile fracture. However, the microcracks tend to nucleate at the σ phase and γ-austenite interface due to inconsistent ductility, which could cause unexpected rapid fracture. The effect of σ phase precipitations on strength is insignificant, but it causes losses in ductility.

## Figures and Tables

**Table 1:** Chemical compositions of as-received 316LN stainless steel (mass%).

C	Si	Mn	P	S	Ni	Cr	Mo	Cu	N	Fe
0.016	0.27	0.81	0.008	0.008	12.12	17.52	2.39	0.08	0.065	Bal.

## Data Availability

Data is contained within the article.
